# Facing High EEG Signals Variability during Classification Using Fractal Dimension and Different Cutoff Frequencies

**DOI:** 10.1155/2019/9174307

**Published:** 2019-05-20

**Authors:** R. Salazar-Varas, Roberto A. Vazquez

**Affiliations:** ^1^Escuela de Ingeniería, Universidad de las Americas Puebla, Sta. Catarina Mártir, Puebla, CP 72810 San Andrés Cholula, Mexico; ^2^Intelligent Systems Group, Facultad de Ingeniería, Universidad La Salle México, Benjamin Franklin 45, Col. Condesa, CP 06140 Mexico City, Mexico

## Abstract

In the development of a brain-computer interface (BCI), some issues should be regarded in order to improve its reliability and performance. Perhaps, one of the most challenging issues is related to the high variability of the brain signals, which directly impacts the accuracy of the classification. In this sense, novel feature extraction techniques should be explored in order to select those able to face this variability. Furthermore, to improve the performance of the selected feature extraction technique, the parameters of the filter applied in the preprocessing stage need to be properly selected. Then, this work presents an analysis of the robustness of the fractal dimension as feature extraction technique under high variability of the EEG signals, particularly when the training data are recorded one day and the testing data are obtained on a different day. The results are compared with those obtained by an autoregressive model, which is a technique commonly used in BCI applications. Also, the effect of properly selecting the cutoff frequencies of the filter in the preprocessing stage is evaluated. This research is supported by several experiments carried out using a public data set from the BCI international competition, specifically data set 2a from BCIIC IV, related to motor tasks. By a statistical test, it is demonstrated that the performance achieved using the fractal dimension is significantly better than that reached by the AR model. Also, it is demonstrated that the selection of the appropriate cutoff frequencies improves significantly the performance in the classification. The increase rate is approximately of 17%.

## 1. Introduction

The performance of any task requires the coordinated activation of a set of neurons. This activity generates bioelectrical signals which can be recorded by the electroencephalography (EEG). The EEG is a recording technique of the brain activity which is noninvasive, of low cost, and provides high time resolution [1].

The information extracted from EEG signals can be useful for different applications such as diagnostics, analysis of the reaction of the brain to any stimulus, or the development of new technologies like brain-computer interface (BCI). A BCI is a system that allows the communication of a subject with a device only through brain activity, without using peripheral nerves [2]. This technology is being applied in different fields such as improvement of concentration, hyperactivity treatment, control of wheel chairs, and spellers [2–4]. The development of this kind of applications requires the following stages: acquisition of the signal, preprocessing, feature extraction, and classification [4]. However, there are different issues that should be addressed in order to achieve reliable and friendly applications that can be used in real life. Some of these challenges are the number of electrodes used in the signal acquisition, the noise sensitivity of the EEG signals, the nonlinearity and nonstationarity of the EEG signals, and the intersubject variability, among others [5, 6]. Perhaps, the most critical challenges are the nonlinearity and nonstationarity of the EEG signals since they are properties of the signal that depends on the organism and on the environment [7–9]. Therefore, it is important to propose different feature extraction techniques that show robustness to perturbations on the signal. In the same way, the intersubject variability is an important challenge since it avoids to develop a generalized BCI. It has been demonstrated that an event-related (de) synchronization in specific frequency bands occurs when a mental task is performed [10]. However, a variability in the EEG signals from different subjects is present due to the anatomical and physiological differences among subjects. Therefore, it is necessary to select an appropriate frequency band for each subject which helps to increase the accuracy in the classification. In this sense, several works have suggested to find the frequency band which improves the performance of the BCI for each subject. Most of these works propose to decompose the EEG signals in different frequency bands. Then, the most suitable band is selected using different techniques, for example, the mutual information computed for the different frequency bands [11–14].

Some feature extraction techniques that have been widely used in the EEG signal analysis are the Fourier transform and the linear prediction. Although these techniques have shown good performance, they do not consider the nonlinearity and nonstarionarity of the EEG signals [15]. In this sense, a nonlinear analysis can provide more information about the signal dynamics related to the physiological phenomenon being explored [15, 16]. Two nonlinear methods which have been commonly used in the signal analysis are the fractal properties and the entropy. They have the ability to express the dynamics of the signal and its complexity [16].

Fractal analysis has been used in the brain signals analysis, providing information about aging, dysfunctions, and response of the brain to the anesthesia [17]. A fractal property commonly used is the fractal dimension (FD), which in the neuroscience field has been widely used for the automatic seizure detection [18, 19]. In the field of the BCIs, the behavior of the FD has been explored under the execution of different mental tasks when (de)synchronization occurs. In this sense, there are reports indicating that the discrimination of different mental tasks is possible using the FD as feature [20–22]. Also, the relation between the FD value computed from the EEG signals and the hand grip force has been explored [23]. Therefore, based on the behavior of the FD during different mental states and the fact that it can be manipulated voluntarily, FD is an attractive candidate to develop BCI applications. However, despite the results obtained from the previous researchers, the estimation of the FD is not a trivial problem that, for that reason, it should be carefully addressed taking into account different conditions such as the window length, the signal to noise ratio, and the autocorrelation. It is well demonstrated that these conditions have different impacts on the algorithms to compute the FD. For example, Katz's method is most consistent in the discrimination of epileptic signals while Higuchi's method provides a more accurate approximation using synthetic signals but is more sensitive to noise [24]. In order to achieve a reliable BCI, it is important to evaluate the robustness of the FD under the high variability of the EEG signals. An extreme situation of this nonstationarity is observed when the EEG recordings are performed on different days. Also, it is necessary to analyze the behavior of the performance of this feature when different frequency bands are used. Thus, this research is focused on demonstrating the ability of the FD in the discrimination of the EEG signals despite the high variability of them. This variability is maximized due to the acquisition on different days. Another point of interest is to show that selecting a correct cutoff frequency can improve the accuracy of the classification.

Therefore, one contribution of this paper is focused on evaluating and analyzing the accuracy of the FD during the classification of different mental tasks employing EEG recordings from different days and a linear discriminant as classifier. The FD can be computed in the time domain or in the phase space [25]. In this research, FD is computed in the time domain by two different methods: Higuchi and Katz. The accuracy achieved by FD is compared with the accuracy obtained with an autoregressive model which is a classical linear technique. Furthermore, a Kruskal–Wallis test was performed in order to evaluate if there exists a significant difference accuracy using the FD.

The second contribution is related to the impact of determining the optimal filter configuration used for each subject in the preprocessing stage. In this sense, we propose to vary the parameters used in the preprocessing, specifically the cutoff frequencies of the bandpass filter. The accuracy reached with the optimal configuration is compared with that obtained when the commonly filter configuration is used (1–100 Hz). Moreover, a hypothesis test was performed in order to evaluate if there is an optimal filter configuration that impacts significantly the classification accuracy.

This paper is organized as follows: in Section 2, a detailed description of the methodology is presented as well as the theoretical concepts that were used.  Section 3 shows a description and discussion of the results. Finally, the conclusions of this paper are presented in Section 4.

## 2. Methods

In this section, we present the methodology proposed to evaluate the robustness of the FD and the impact in the accuracy when an optimal filter configuration is selected. This methodology was segmented in the classical BCI stages: preprocessing, feature extraction, and classification. The proposal in each stage is explained in the following.

### 2.1. Preprocessing

In this stage, the window length and the filter configuration were analyzed. Firstly, to assess the window length impact on the performance of the fractal dimension during classification task, we evaluate three different lengths: 1 s, 1.5 s, and 2 s. After that, different cutoff frequencies were applied in order to select the values which help us to improve the results in the classification. The parameters were fixed as follows: the value of the low cutoff frequency varies from 1 to 125 *W* in steps of one, where *W* represents the width of the passband and its value is varied from 10 to 100 with increases of 10.

### 2.2. Feature Extraction

Once the signal has been preprocessed, the next stage consists in to extract descriptive information of the signal to generate a feature vector *y*. Then, considering the brain activity is recorded with a set of *M* electrodes, a feature vector *y* is computed by the features extracted from each one of the *M* electrodes, using the technique selected for this goal. The complexity and the dynamics of a signal can be analyzed through different nonlinear methods, like its fractal properties. A fractal property commonly used is the FD, and it is computed in the time domain and in the phase space. In this research, the FD is computed in the time domain by the most known methods: Higuchi and Katz. In addition, FD is compared against AR coefficients [26]. The feature vector was *M*-dimensional when the FD was used and *p∗M*-dimensional for the AR model, where *M* expresses the number of channels and *p* indicates the order of the AR model.

### 2.3. Classification

In this stage, a linear discriminant was used. In order to evaluate the robustness of each feature, the classifier was trained with a data set recorded on one day and the evaluation of the classification was performed using the data recorded on a different day. Focused on performing a statistical test, the training and evaluation stages were done several times with subdata sets generated randomly from original data sets.

## 3. Results and Discussion

In this section, the proposed methodology is evaluated to assess its robustness under the variability of the EEG signals recorded on different days. The experimental results were obtained using the database 2a from the BCI International competition IV. This data set is made up of four imaginary motor tasks executed during three seconds: left hand, right hand, both feet, and tongue. For this research, a two-class discrimination task was performed using two different combinations: left vs right hand and both feet vs tongue. A set of 22 electrodes was used to record the EEG signals, and the sampling frequency was 250 Hz.

The data set provides the recordings of nine subjects. For each subject, two sessions were performed on different days, recording six runs for each one. One run is composed by 12 trials for each mental task (i.e., 48 trials per run).

The classification was performed in two different conditions:

- *Condition A.* The data recorded during the first session were employed to train the classifier, and it was tested using the data recorded during the second session.
- *Condition B.* The classifier was trained with the data recorded during the second session, and the data recorded during the first session were employed to test the classifier.

In order to obtain statistically significant results, we performed 30 experiments using training and testing subsets that were randomly built using 70% of the trails from the original data sets. This percentage was fixed regarding the minimum number of trials necessary to train the classifier, given the dimension of the feature vector.

Before starting the analysis, we evaluate the impact of the window length in the accuracy of the FD during the classification task. To this goal, three different lengths were used: 1 s, 1.5 s, and 2 s. In order to evaluate all the possible scenarios using different window lengths, we take into account *Condition A* and *Condition B*. For both conditions, the data were filtered using the classical cutoff frequencies and the optimal cutoff frequencies for each subject.

We compare the average accuracy for each feature extraction technique. Although slight differences were found, these differences are not statistically significant. Even so, in most of the cases, the highest accuracy was achieved when a windows length of 2 s was employed. Therefore, this length was selected.

The results obtained for *Condition A* filtering the signal with the classical cutoff frequencies (1–100 Hz) are shown in Table 1. For each subject, the result in bold is the best average accuracy achieved from the different feature extraction techniques. In order to evaluate if a significant difference exists among these accuracy values, a Kruskal–Wallis test was applied. In the cases where a significant difference was found, a multicomparison test was performed. The asterisks in Table 1 indicate the accuracies that are significantly different compared with the best result.

As can be observed, the best results were obtained mostly when the fractal dimension is used as feature extraction technique. A significant difference is also observed in the comparison of the best accuracy obtained by the FD technique against that obtained by AR. Furthermore, for most of the cases where the best results were obtained using AR, the accuracy was close to the random level and in few cases, the difference with the other techniques is significant. It is important to notice that only for three subjects, an accuracy greater than 70% was obtained.

**Figure 1:**
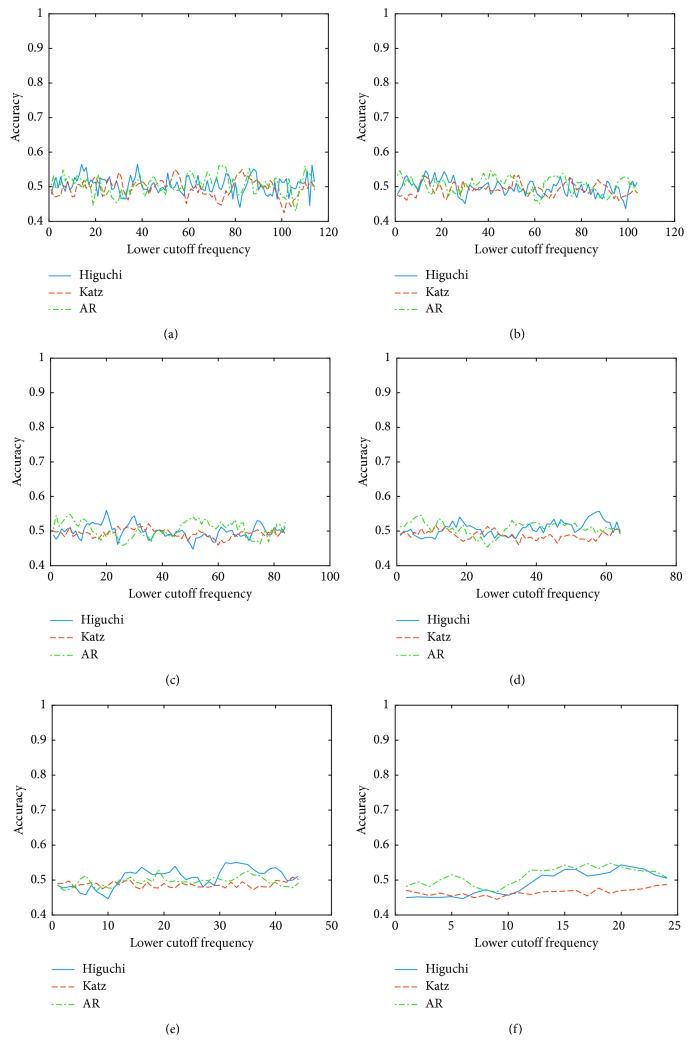
Accuracy using different cutoff frequencies for the worst subject for *Condition A*. (a) Width of the passband 10 Hz. (b) Width of the passband 20 Hz. (c) Width of the passband 40 Hz. (d) Width of the passband 60 Hz. (e) Width of the passband 80 Hz. (f) Width of the passband 100 Hz.

**Figure 2:**
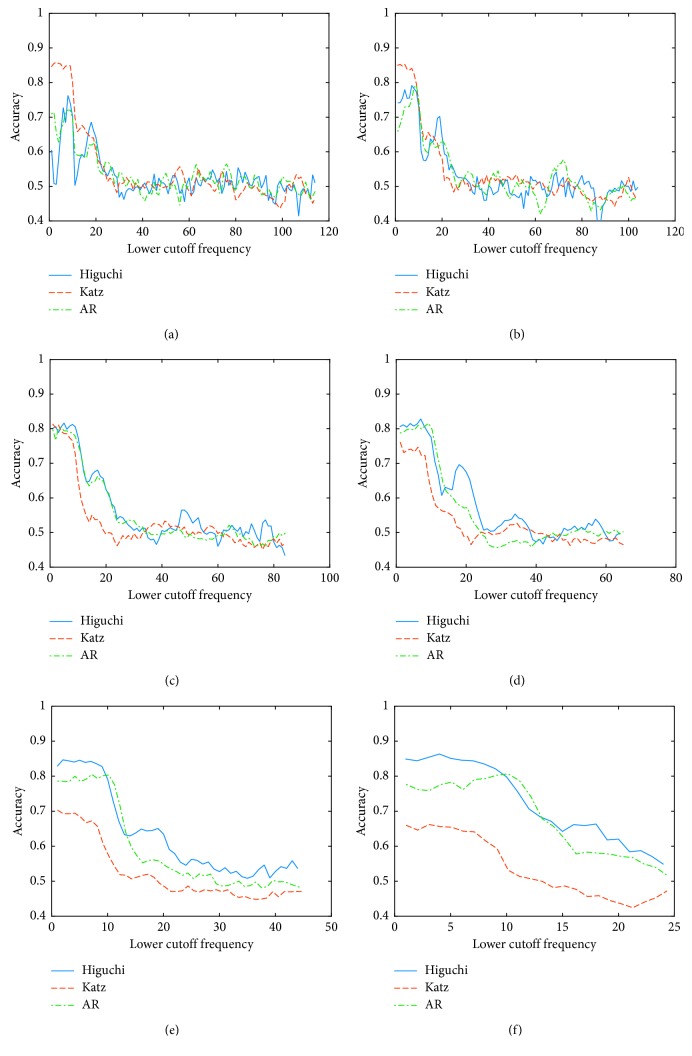
Accuracy using different cutoff frequencies for the best subject for *Condition A*. (a) Width of the passband 10 Hz. (b) Width of the passband 20 Hz. (c) Width of the passband 40 Hz. (d) Width of the passband 60 Hz. (e) Width of the passband 80 Hz. (f) Width of the passband 100 Hz.

The second analysis was focused on the accuracy using different cutoff frequencies, in order to determine the best filter configuration (bandwidth and low cutoff frequency) considering the intersubject variability. Figures 1 and 2 show the results of this analysis for the worst (A4) and best (A8) subject, respectively. Each plot shows the accuracy for the different feature extraction techniques using a specific bandwidth; the *x* axis indicates the low cutoff frequency, and the *y* axis corresponds to the accuracy obtained for that filter configuration.

Analyzing the graphical results for the worst subject, it is possible to say that although there are some configurations that slightly improve the accuracy, for most of the cases, the improvement was not enough to surpass the minimum level of randomness. Nonetheless, in the case of the best subject, the best results are obtained when low cutoff frequencies are used. Considering these low frequencies, it is important to carefully select the bandwidth depending on the feature extraction technique. However, the best results are obtained using a narrow bandpass for Katz's method; Higuchi and AR methods provide better results using a wide bandpass. Furthermore, the best results are obtained when Higuchi's method is used as feature extraction technique and with the following filter configuration: low cutoff frequency = 4 Hz; width of the passband (*W*) = 100 Hz.  Table 2 shows the cutoff frequencies that provide the best accuracy for each subject and each feature extraction technique. It is important to remark that the selected frequencies are different to the frequencies commonly used in the BCI applications.

The results obtained with the selected configuration are shown in Table 3. For each subject, the accuracy achieved with each feature extraction technique is displayed and the maximum value is in bold. As in the previous case, a Kruskal–Wallis test was applied in order to know if there is a significant difference among the accuracy values. The asterisks in Table 3 indicate a significant difference between the value and the best accuracy.

Table 4 shows the increase rate achieved using the selected filter configuration. As can be observed, the improvement in most of the cases is higher than 10% with respect to the accuracy obtained using the classical filter configuration (1–100 Hz). In order to confirm if this improvement is statistically significant, a hypothesis test with a significance level of 0.01 was applied. In most of the cases, this difference was significant.

The same analysis was applied for *Condition B*, when the classifier was trained with the data recorded on the second day and was tested with the data from the first day.

The accuracies obtained for *Condition B* using the classical cutoff frequencies (1–100 Hz) are shown in Table 5. The best accuracy for each subject is in bold. As in *Condition A*, in most of the cases, the best results are achieved when the FD is used as feature extraction technique.

A Kruskal–Wallis test was applied to assess if there is any significant difference. For the cases with significant difference, a multicomparison test was applied. The results are indicated with the asterisks in Table 5.

**Figure 3:**
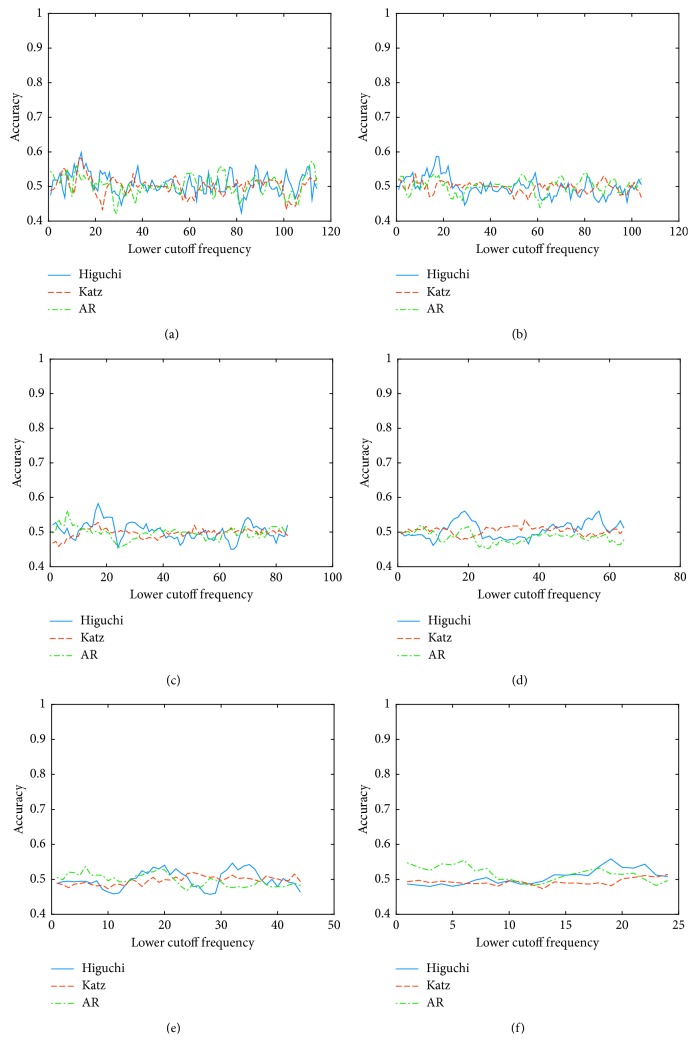
Accuracy using different cutoff frequencies for the worst subject for *Condition B*. (a) Width of the passband 10 Hz. (b) Width of the passband 20 Hz. (c) Width of the passband 40 Hz. (d) Width of the passband 60 Hz. (e) Width of the passband 80 Hz. (f) Width of the passband 100 Hz.

**Figure 4:**
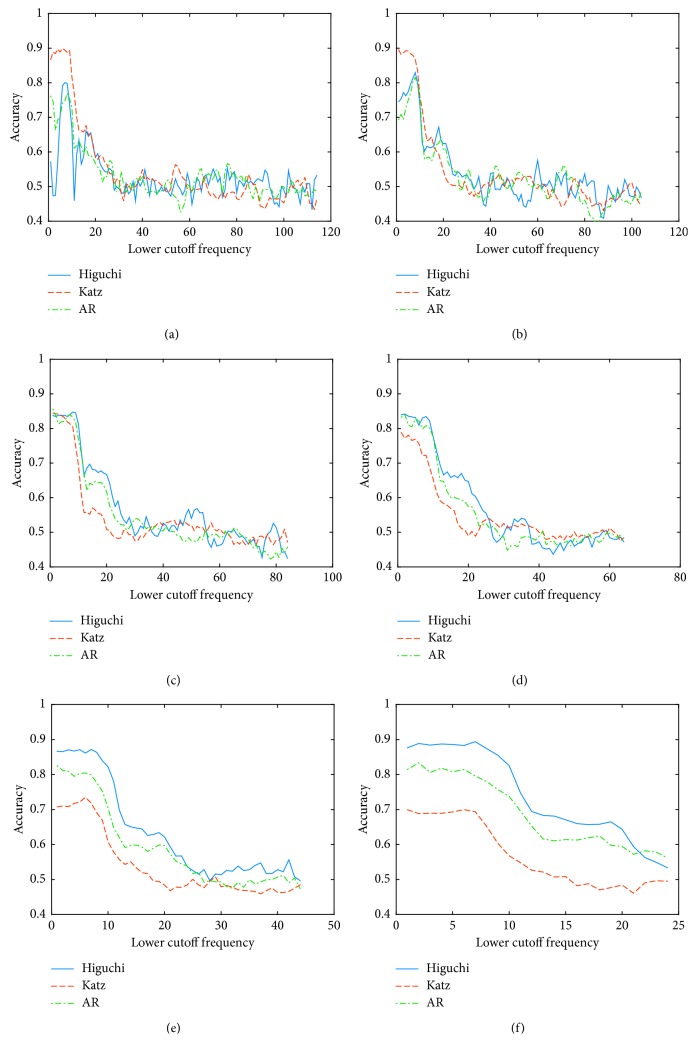
Accuracy using different cutoff frequencies for the best subject for *Condition B*. (a) Width of the passband 10 Hz. (b) Width of the passband 20 Hz. (c) Width of the passband 40 Hz. (d) Width of the passband 60 Hz. (e) Width of the passband 80 Hz. (f) Width of the passband 100 Hz.

Following with the study, the behavior of the accuracy was analyzed using different filter configurations. The graphical results for the worst (A4) and best (A8) subjects are shown in Figures 3 and 4, respectively. Once more, for the best subject, it is notorious that the accuracy depends on the cutoff frequencies used in the preprocessing stage. Based on this analysis, the selected frequencies for each subject and each feature extraction technique are shown in Table 6.

Once the most appropriated cutoff frequencies were selected, the accuracies using the different feature extraction techniques were computed (Table 7). Once more, it can be seen that the feature extraction technique that provides the best results is the FD, mainly Higuchi's method. The significant differences found through a multicomparison test are indicated with asterisks in Table 7.

The increasing rates achieved using the selected filter configuration are shown in Table 8. Based on these results, it is possible to say that, as in *Condition A*, an adequate selection of the cutoff frequencies has a positive impact in the performance of the classification task, and this improvement is present for all feature extraction techniques.

Furthermore, the improvement achieved on the accuracy when the cutoff frequencies are carefully selected is statistically significant with a significance level of 0.01.

Finally, the proposed methodology was applied to the data recorded when the subject performs less-common imaginary motor tasks. The movements imagined by the subjects were the feet and tongue. The first analysis was to select the most suitable window length. In this case, when the fractal dimension is used as feature extraction technique for some subjects, the use of 1 s length is better than 2 s, and for other subjects, the best results are obtained using 2 s length. In the case of AR, for most of the subjects, the best length is 2 s. In the comparison of the window lengths for the same condition but filtering the data with the optimal cutoff frequencies, for all the feature extraction techniques, the accuracy is higher when the window length is 2 s. For the case of *Condition B*, it is not possible to say that there are better results with a specific window length used. Finally, using the best cutoff frequencies for each subject during *Condition B*, for most of the subjects, the best results are obtained using a window length of 2 s.

Based on the described observations, we selected a 2 s window length since for most of the experiments, this length provides the best results.

Under these conditions, Table 9 shows the accuracies for the three feature extraction techniques. The maximum value is in bold, and the asterisks indicate a significant difference with the maximum value. As it can be seen, for most of the subjects, the accuracy is close to randomness; this could be caused by the fact that the subjects are less familiarized with these movements. On the other hand, for most of the subjects, the best results are achieved when FD is used as feature.

Following the methodology, we achieved the analysis of the accuracy varying the cutoff frequencies. In Table 10 are shown the frequencies selected for each subject and each technique. Using these cutoff frequencies, the results of the classification are shown in Table 11. As it can be seen, the increase achieved with the frequencies selection is highly noticeable. For most of the subjects, the best results are achieved by Katz's method and in most of the cases, the improvement was significant. The increase rate is shown in Table 12.

Finally, we analyzed *Condition B* using this movement combination. As in *Condition A*, using the classical filter configuration, the accuracies are close to randomness (Table 13), and the higher results are obtained when the FD is used as feature.

As equal as in the previous experiments, we selected the best cutoff frequencies for each subject; these frequencies are listed in Table 14. Using these frequencies, it was possible to improve the results considerable, as is reported in Table 15. Furthermore, this increase is statistically significant for most of the cases.

Finally, the increase rate is reported in Table 16.

Based on this analysis, considering both conditions and the different mental tasks performed by the subjects, we can observe that the selection of appropriate cutoff frequencies highly impacts the accuracy of a BCI application. This impact was more notorious with the less-common movements (tongue and feet).

It is also important to mention that the window length has an effect in the accuracy as well. Based on the experimental results, we observed that the best accuracy is obtained in general with a 2 s window length, but in other cases, better results are obtained with different window lengths. Therefore, the results could be improved if a suitable window length is selected for each subject.

## 4. Conclusions

This paper presented a careful evaluation of the robustness of the fractal dimension (FD) as feature extraction technique in the classification of the EEG signals under high variability conditions, particularly when training and testing data sets are recorded on different days. This problem represents a crucial challenge in the BCI applications due to the nonstationarity of the EEG signals. In order to assess the accuracy of the proposed methodology, we use the data set 2a from BCIIC IV under two conditions. Firstly, the classifier was trained with the data from the first day and was tested using the data from the second day. For the other condition, the data from the second day were used to train the classifier and the data from the first session were used to test it.

In the preprocessing stage, different windows lengths (1 s, 1.5 s, and 2 s) were evaluated in order to select the most suitable. Although in most of the cases, a higher accuracy was obtained when a window length of 2 s was employed, for some cases, the highest accuracy was achieved with other windows length. Therefore, a detailed analysis for each subject is recommended in order to achieve the most reliable BCI in each case. After the selection of window length, the EEG signals were filtered applying a bandpass filter with 1–100 Hz as cutoff frequencies. To generate the feature vector, the FD was computed in the time domain by two different methods: Higuchi and Katz. In order to know if FD provides better results than those obtained with classical feature extraction techniques, the performance achieved with FD was compared with that reached when an AR model of second order is used. For most of the cases, the results obtained by the FD (computed with the Higuchi or Katz method) are better than those obtained using the AR model. Additionally, through a Kruskal–Wallis test and a multicomparison test was shown that several of these differences are significant.

Furthermore, the impact of selecting the cutoff frequencies for each subject in the preprocessing stage was analyzed as well. For this goal, the low cutoff frequency and the width of passband of the filter used in the preprocessing stage were varied. By the obtained results was demonstrated that the frequency selection allows an average increase for both conditions close to 18% and this increase is significant for most of the subjects for the three evaluated techniques. Particularly for Higuchi and Katz, we obtained an average increase of 18% and 21%, respectively. The methodology was also applied during the classification of two movements less common; in this case, the improvement of the accuracy due to the frequencies selection was more remarkable.

The obtained results suggest that it is possible to design a robust BCI able to face the nonstationarity of the EEG signals whose performance can be improved by selecting the most appropriate cutoff frequencies. Nowadays, we are evaluating the performance of the FD using different classifiers such as spiking neural networks in order to improve the obtained results. Furthermore, we are working to establish a robust methodology to develop a BCI based on FD and spiking neural networks.

## Figures and Tables

**Table 1:** Average accuracy for *Condition A* using the classical filter configuration (1–100 Hz).

Subject	Higuchi	Katz	AR
A1	**0.65** ± **0.04**	0.58 ± 0.03^*∗*^	0.61 ± 0.04^*∗*^
A2	0.49 ± 0.05^*∗*^	0.51 ± 0.02	**0.52** ± **0.03**
A3	**0.78** ± **0.03**	0.51 ± 0.05^*∗*^	0.65 ± 0.04^*∗*^
A4	0.45 ± 0.04^*∗*^	0.47 ± 0.03	**0.48** ± **0.04**
A5	**0.54** ± **0.05**	0.51 ± 0.03^*∗*^	0.51 ± 0.03^*∗*^
A6	0.54 ± 0.04	**0.55** ± **0.03**	0.53 ± 0.05
A7	0.53 ± 0.04^*∗*^	**0.55** ± **0.04**	**0.55** ± **0.04**
A8	**0.85** ± **0.03**	0.66 ± 0.04^*∗*^	0.78 ± 0.04^*∗*^
A9	0.65 ± 0.05^*∗*^	**0.78** ± **0.07**	0.69 ± 0.06^*∗*^
**Average**	**0.61** ± **0.04**	0.57 ± 0.04	0.59 ± 0.04

^*∗*^Accuracies that are significantly different compared with the best result. Values in bold indicate the best average accuracy achieved from the different feature extraction techniques.

**Table 2:** Cutoff frequencies selected for each subject for *Condition A*.

Subject	Higuchi	Katz	AR
A1	9–109	9–29	3–13
A2	34–44	13–23	22–32
A3	10–20	4–14	10–40
A4	38–48	83–93	58–88
A5	5–35	25–35	33–43
A6	17–37	24–34	2–32
A7	3–23	2–32	2–42
A8	4–104	3–13	10–80
A9	26–36	4–64	14–94

**Table 3:** Average accuracy for *Condition A* using the selected filter configuration.

Subject	Higuchi	Katz	AR
A1	0.68 ± 0.04	**0.70** ± **0.04**	0.66 ± 0.05^*∗*^
A2	0.57 ± 0.03	0.57 ± 0.05	**0.58** ± **0.04**
A3	**0.83** ± **0.03**	0.82 ± 0.04	0.77 ± 0.04^*∗*^
A4	**0.57** ± **0.04**	0.55 ± 0.03	0.56 ± 0.04
A5	**0.62** ± **0.03**	0.57 ± 0.03^*∗*^	0.57 ± 0.05^*∗*^
A6	0.58 ± 0.04^*∗*^	**0.63** ± **0.05**	0.59 ± 0.03^*∗*^
A7	0.61 ± 0.04^*∗*^	**0.65** ± **0.05**	0.61 ± 0.04^*∗*^
A8	**0.86** ± **0.03**	**0.86** ± **0.03**	0.81 ± 0.04^*∗*^
A9	0.82 ± 0.03	**0.83** ± **0.06**	0.80 ± 0.06
**Average**	0.68 ± 0.03	**0.69** ± **0.04**	0.66 ± 0.04

^*∗*^Accuracies that are significantly different compared with the best result. Values in bold indicate the best average accuracy achieved from the different feature extraction techniques.

**Table 4:** Increase rate for *Condition A*.

Subject	Higuchi	Katz	AR
A1	4.91	21.41	7.17
A2	15.67	12.38	12.02
A3	7.70	61.42	17.12
A4	25.72	16.86	17.01
A5	14.50	11.92	9.92
A6	8.47	14.93	11.23
A7	15.23	18.56	10.39
A8	1.69	30.23	4.42
A9	27.08	6.13	15.42
**Average**	13.44	21.53	11.63

**Table 5:** Average accuracy for *Condition B* using the classical filter configuration (1–100 Hz).

Subject	Higuchi	Katz	AR
A1	0.59 ± 0.06^*∗*^	0.55 ± 0.04^*∗*^	**0.66** ± **0.04**
A2	**0.55** ± **0.03**	0.49 ± 0.04^*∗*^	**0.55** ± **0.05**
A3	**0.80** ± **0.05**	0.53 ± 0.05^*∗*^	0.65 ± 0.04^*∗*^
A4	0.49 ± 0.03^*∗*^	0.49 ± 0.02^*∗*^	**0.55** ± **0.04**
A5	0.52 ± 0.04	0.51 ± 0.03^*∗*^	**0.53** ± **0.04**
A6	0.58 ± 0.04^*∗*^	**0.61** ± **0.04**	0.54 ± 0.04^*∗*^
A7	0.52 ± 0.02	**0.53** ± **0.02**	0.52 ± 0.03
A8	**0.88** ± **0.03**	0.70 ± 0.04^*∗*^	0.81 ± 0.04^*∗*^
A9	0.62 ± 0.04^*∗*^	**0.74** ± **0.07**	0.63 ± 0.08^*∗*^
**Average**	**0.62** ± **0.04**	0.57 ± 0.04	0.60 ± 0.04

^*∗*^Accuracies that are significantly different compared with the best result. Values in bold indicate the best average accuracy achieved from the different feature extraction techniques.

**Table 6:** Cutoff frequencies selected for each subject for *Condition B*.

Subject	Higuchi	Katz	AR
A1	9–29	18–28	2–82
A2	9–39	25–45	4–84
A3	8–48	7–17	8–48
A4	14–24	14–24	112–122
A5	5–35	27–37	16–36
A6	8–68	10–90	4–34
A7	3–23	6–16	3–23
A8	7–107	6–16	1–41
A9	26–36	9–19	2–12

**Table 7:** Average accuracy for *Condition B* using the selected filter configuration.

Subject	Higuchi	Katz	AR
A1	0.69 ± 0.03	**0.71** ± **0.03**	0.69 ± 0.03
A2	**0.60** ± **0.03**	0.58 ± 0.03^*∗*^	**0.60** ± **0.04**
A3	**0.87** ± **0.03**	0.83 ± 0.04^*∗*^	0.83 ± 0.04^*∗*^
A4	**0.60** ± **0.05**	0.58 ± 0.03	0.57 ± 0.05
A5	**0.68** ± **0.04**	0.67 ± 0.04	0.64 ± 0.03^*∗*^
A6	0.64 ± 0.03	**0.65** ± **0.04**	0.61 ± 0.04^*∗*^
A7	**0.64** ± **0.03**	0.63 ± 0.05	0.62 ± 0.05
A8	0.89 ± 0.04	**0.90** ± **0.03**	0.86 ± 0.03^*∗*^
A9	0.82 ± 0.04^*∗*^	**0.88** ± **0.03**	0.82 ± 0.03^*∗*^
**Average**	**0.71** ± **0.04**	**0.71** ± **0.04**	0.69 ± 0.04

^*∗*^Accuracies that are significantly different compared with the best result. Values in bold indicate the best average accuracy achieved from the different feature extraction techniques.

**Table 8:** Increase rate for *Condition B*.

Subject	Higuchi	Katz	AR
A1	16.12	27.67	3.56
A2	9.35	16.56	8.79
A3	7.68	56.52	27.48
A4	22.93	17.99	4.82
A5	30.71	30.95	20.79
A6	8.85	5.57	13.26
A7	22.10	19.02	20.90
A8	1.98	28.60	5.02
A9	32.99	18.78	31.41
**Average**	16.97	24.62	15.11

**Table 9:** Average accuracy for *Condition A* during the classification of feet and tongue movements and using the classical filter configuration (1–100).

Subject	Higuchi	Katz	AR
A1	0.55 ± 0.04^*∗*^	0.55 ± 0.04^*∗*^	**0.58** ± **0.03**^*∗*^
A2	0.52 ± 0.04	**0.55** ± **0.03**	0.54 ± 0.04
A3	**0.64** ± **0.03**	0.58 ± 0.04^*∗*^	0.62 ± 0.05
A4	0.48 ± 0.04^*∗*^	**0.52** ± **0.03**	0.50 ± 0.03
A5	**0.53** ± **0.04**	0.50 ± 0.02^*∗*^	0.50 ± 0.01^*∗*^
A6	0.54 ± 0.04^*∗*^	**0.57** ± **0.03**	0.54 ± 0.04^*∗*^
A7	0.54 ± 0.03^*∗*^	0.59 ± 0.06^*∗*^	**0.64** ± **0.05**
A8	**0.73** ± **0.03**	0.68 ± 0.04^*∗*^	0.62 ± 0.05^*∗*^
A9	0.68 ± 0.04^*∗*^	**0.75** ± **0.06**	0.70 ± 0.06^*∗*^
**Average**	0.58 ± 0.04	**0.59** ± **0.04**	0.58 ± 0.04

^*∗*^Accuracies that are significantly different compared with the best result. Values in bold indicate the best average accuracy achieved from the different feature extraction techniques.

**Table 10:** Cutoff frequencies selected for each subject for *Condition A* during the classification of feet and tongue movements.

Subject	Higuchi	Katz	AR
A1	22–102	10–20	11–101
A2	3–33	7–37	13–33
A3	11–61	10–20	12–52
A4	85–105	71–81	78–98
A5	8–18	8–38	14–24
A6	10–20	2–32	12–42
A7	23–103	21–31	3–33
A8	8–38	3–23	8–98
A9	6–96	7–27	6–96

**Table 11:** Average accuracy for *Condition A* during the classification of feet and tongue movements and using the optimal filter configuration.

Subject	Higuchi	Katz	AR
A1	0.65 ± 0.04^*∗*^	**0.67** ± **0.03**	0.62 ± 0.04^*∗*^
A2	0.63 ± 0.04	**0.65** ± **0.06**	0.60 ± 0.04^*∗*^
A3	0.74 ± 0.03^*∗*^	**0.79** ± **0.03**	0.70 ± 0.04^*∗*^
A4	0.56 ± 0.03	0.56 ± 0.05	0.56 ± 0.04
A5	**0.60** ± **0.05**	0.59 ± 0.04	0.57 ± 0.05^*∗*^
A6	0.64 ± 0.03^*∗*^	**0.68** ± **0.05**	0.59 ± 0.05^*∗*^
A7	0.73 ± 0.04^*∗*^	**0.78** ± **0.04**	0.72 ± 0.05^*∗*^
A8	0.74 ± 0.03^*∗*^	**0.78** ± **0.04**	0.72 ± 0.02^*∗*^
A9	0.74 ± 0.06^*∗*^	**0.84** ± **0.03**	0.73 ± 0.06^*∗*^
**Average**	0.67 ± 0.04	**0.70** ± **0.04**	0.65 ± 0.04

^*∗*^Accuracies that are significantly different compared with the best result. Values in bold indicate the best average accuracy achieved from the different feature extraction techniques.

**Table 12:** Increase rate for *Condition A* during the classification of feet and tongue movements.

Subject	Higuchi	Katz	AR
A1	16.91	22.72	5.70
A2	19.34	18.69	12.41
A3	14.71	37.13	11.85
A4	16.08	9.51	12.41
A5	12.67	17.26	13.04
A6	18.47	19.98	8.80
A7	33.93	31.25	13.06
A8	0.91	14.18	15.32
A9	8.89	11.45	4.59
**Average**	15.77	20.24	10.80

**Table 13:** Average accuracy for *Condition B* during the classification of feet and tongue movements and using the classical filter configuration (1–100).

Subject	Higuchi	Katz	AR
A1	0.55 ± 0.03^*∗*^	0.57 ± 0.04^*∗*^	**0.62** ± **0.04**
A2	0.51 ± 0.03^*∗*^	**0.59** ± **0.05**	0.51 ± 0.03^*∗*^
A3	**0.62** ± **0.03**	0.55 ± 0.04^*∗*^	0.54 ± 0.05^*∗*^
A4	0.50 ± 0.03	0.50 ± 0.04	**0.51** ± **0.03**
A5	**0.54** ± **0.05**	0.51 ± 0.02^*∗*^	0.50 ± 0.02^*∗*^
A6	0.58 ± 0.05	**0.59** ± **0.03**	0.55 ± 0.04^*∗*^
A7	0.60 ± 0.05	**0.62** ± **0.07**	0.60 ± 0.07
A8	**0.73** ± **0.04**	0.72 ± 0.05	0.64 ± 0.05^*∗*^
A9	0.56 ± 0.03^*∗*^	**0.71** ± **0.05**	0.65 ± 0.06^*∗*^
**Average**	0.58 ± 0.04	**0.60** ± **0.04**	0.57 ± 0.04

^*∗*^Accuracies that are significantly different compared with the best result. Values in bold indicate the best average accuracy achieved from the different feature extraction techniques.

**Table 14:** Cutoff frequencies selected for each subject for *Condition B* during the classification of feet and tongue movements.

Subject	Higuchi	Katz	AR
A1	11–21	8–18	10–100
A2	11–31	21–31	25–45
A3	9–39	10–20	11–41
A4	95–115	58–68	54–64
A5	18–118	13–33	15–35
A6	11–61	13–23	17–47
A7	1–31	16–36	8–28
A8	5–35	13–23	1–41
A9	9–49	9–19	6–46

**Table 15:** Average accuracy for *Condition B* during the classification of feet and tongue movements and using the optimal filter configuration.

Subject	Higuchi	Katz	AR
A1	0.67 ± 0.03^*∗*^	**0.78** ± **0.04**	0.65 ± 0.04^*∗*^
A2	0.67 ± 0.05	**0.69** ± **0.05**	0.61 ± 0.05^*∗*^
A3	**0.76** ± **0.03**	0.74 ± 0.04	0.73 ± 0.06^*∗*^
A4	0.57 ± 0.04	0.56 ± 0.04	**0.58** ± **0.04**
A5	0.63 ± 0.05	**0.64** ± **0.04**	0.61 ± 0.05^*∗*^
A6	0.65 ± 0.05	**0.66** ± **0.05**	0.61 ± 0.05^*∗*^
A7	0.74 ± 0.04^*∗*^	**0.82** ± **0.04**	0.70 ± 0.04^*∗*^
A8	0.77 ± 0.03	**0.79** ± **0.03**	0.74 ± 0.05^*∗*^
A9	0.78 ± 0.04^*∗*^	**0.83** ± **0.03**	0.74 ± 0.04^*∗*^
**Average**	0.69 ± 0.04	**0.72** ± **0.04**	0.66 ± 0.05

^*∗*^Accuracies that are significantly different compared with the best result. Values in bold indicate the best average accuracy achieved from the different feature extraction techniques.

**Table 16:** Increase rate for *Condition B* during the classification of feet and tongue movements.

Subject	Higuchi	Katz	AR
A1	20.38	35.61	3.48
A2	32.64	18.23	19.91
A3	21.69	33.40	34.34
A4	14.70	13.05	12.11
A5	16.34	27.27	21.69
A6	10.88	11.21	11.48
A7	24.01	33.06	16.78
A8	5.80	8.59	16.51
A9	39.13	15.80	13.17
**Average**	20.62	21.80	16.61

## Data Availability

The data used to support the findings of this study are available at http://www.bbci.de/competition/.
